# Pharmacogenomic predictors of drug response and choice in dyslipidemia and hypertension

**DOI:** 10.64898/2026.01.28.26345024

**Published:** 2026-01-30

**Authors:** Fumihiko Takeuchi, Malathi S.I. Dona, William W.H. Ho, Samuel A. Lambert, Michael Inouye, Norihiro Kato

**Affiliations:** a Department of Bioinformatics, National Institute of Global Health and Medicine, Japan Institute for Health Security, Tokyo, Japan; b Cambridge Baker Systems Genomics Initiative, Baker Heart and Diabetes Institute, Melbourne, Australia; c Baker Department of Cardiometabolic Health, Melbourne Medical School, The University of Melbourne, Melbourne, Australia.; d Baker Department of Cardiovascular Research, Translation and Implementation, La Trobe University, Melbourne, Australia.; e Cambridge Baker Systems Genomics Initiative, Department of Public Health and Primary Care, University of Cambridge, Cambridge, UK; f British Heart Foundation Cardiovascular Epidemiology Unit, Department of Public Health and Primary Care, University of Cambridge, Cambridge, UK; g Victor Phillip Dahdaleh Heart and Lung Research Institute, University of Cambridge, Cambridge, UK; h Health Data Research UK Cambridge, Wellcome Genome Campus and University of Cambridge, Cambridge, UK; i British Heart Foundation Centre of Research Excellence, University of Cambridge, Cambridge, UK; j Medical Genomics Center, National Institute of Global Health and Medicine, Japan Institute for Health Security, Tokyo, Japan

**Keywords:** Box-Cox transformation, cardiometabolic traits, drug choice, drug efficacy, dyslipidemia, genome-wide association study, hypertension, pharmacogenomics, polygenic score

## Abstract

**Background:**

Drug suitability is determined by safety, efficacy, and pathological appropriateness. The pharmacogenomics of drug suitability can be assessed by analyzing drug response and drug choice in large population cohorts.

**Methods:**

We investigated drug response and drug choice for dyslipidemia and hypertension using genetic, phenotypic, and prescribing data from the UK Biobank and the All of Us Research Program. Drug response was reassessed with rigorous biomarker scaling, while genome-wide association studies (GWAS) and polygenic scores were used to examine genetic factors influencing drug choice.

**Results:**

Conventional analyses showed that variants influencing baseline LDL cholesterol (LDL-C) were inversely associated with absolute LDL-C change but concordant with relative change following statin therapy; these signals disappeared after applying a variance-stabilizing Box–Cox transformation, indicating a methodological artifact in biomarker scaling. GWAS for drug choice identified several significant loci and unique genetic correlation patterns with cardiometabolic traits. Polygenic scores for drug choice yielded statistically significant predictive performance, which was enhanced by incorporating demographic factors, though prediction strength in clinical settings remains modest.

**Conclusion:**

Variance-stabilizing transformation corrects spurious pharmacogenetic associations introduced by biomarker scaling. Genetic variation informs drug choice for dyslipidemia and hypertension, but current polygenic scores provide only modest benefits in clinical application.

## 1. Introduction

Drug suitability for a patient depends on safety, efficacy, and pathological appropriateness. This study focuses on the pharmacogenomic determinants of efficacy and overall suitability by investigating drug response and drug choice for dyslipidemia and hypertension in the UK Biobank and the All of Us Research Program.

Pharmacogenomics has characterized the genetic determinants of drug efficacy, particularly in relation to LDL cholesterol (LDL-C) response to statins. Several variants in *LPA*, *APOE*, *SORT1*, and *SLCO1B1* genes influence statin response [1,2], and most of these variants are also associated with baseline LDL-C levels [3]. In contrast, genome-wide association studies (GWAS) on blood pressure response to antihypertensive medications have not yet identified any loci reaching genome-wide significance [4,5].

In replicating prior GWAS on statin response, we analyze both absolute and relative LDL-C changes as phenotypes, following conventional methodologies. However, our findings suggest that previously reported associations may be artifacts introduced by the scaling methods: raw scale for absolute change and log-scale for relative change. Notably, when employing a variance-stabilizing transformation, which serves as an intermediate approach between these two scales, the previously observed association signals disappear.

Drug selection refers to the process of choosing specific drugs for a particular patient population based on scientific evaluations of their safety, clinical efficacy, and pathological appropriateness. Outside oncology, drug selection based on genetic factors remains uncommon, as disease subclassification primarily rely on clinical measurements. Optimal drug treatment for cardiometabolic diseases such as dyslipidemia and hypertension requires both the appropriate type (or class) for the target disease and the appropriate dosage for the individual patient. Cholesterol medications, including statin and ezetimibe for lowering LDL-C and previously fibrate for lowering triglycerides, are prescribed when cardiovascular risk becomes elevated (https://www.nice.org.uk/guidance/ng238). For hypertension, first-line treatment typically includes diuretics, calcium channel blockers or renin-angiotensin system (RAS)-acting agents (https://www.nice.org.uk/guidance/NG136, https://www.who.int/publications/i/item/9789240033986). If blood pressure remains uncontrolled, dose escalation or addition of β-blockers follows. Even when standard medications and dosages are administered according to clinical guidelines, considerable interindividual variation exists in both efficacy and risk of adverse events. The drug selection process considers comorbidities, age, ethnicity and biomarker responses observed during titration, which can extend over several months.

This study aims to integrate genetic information into the drug selection process. We envision a scenario in which a newly diagnosed dyslipidemic or hypertensive patient receives a drug recommendation informed by their genomic profile. Our underlying hypothesis is that prescription patterns in large biobanks reveal genome-prescription associations that can inform future drug choices. The trait of interest is the choice of drug *X* for disease *Y*, defining cases as patients prescribed drug *X* for disease *Y* and controls as disease *Y* patients not prescribed drug *X*. As we utilize real-world prescription data, the observed drug choice reflects the disease severity, comorbidities, and prior drug response. This “drug choice” trait differs from the previously studied “drug use” trait, which defined controls as individuals not prescribed the drug regardless of disease status [6]. We conduct pharmacogenomic analyses of three types of dyslipidemia treatments (statin, fibrate, and ezetimibe) and four classes of antihypertensive drugs (diuretic, β-blocker, calcium channel blocker, and RAS-acting agents) using genetic and phenotypic data collected from large general population cohorts. We examine the genetic basis of drug choice and assess the feasibility of drug recommendation using germline genetic variation by developing polygenic scores (PGS) for drug choice. This drug choice PGS exhibited modest but statistically significant predictability regarding the appropriateness of prescribing specific drug classes to a given patient population.

## 2. Methods

### 2.1. UK Biobank

The UK Biobank is a large-scale biomedical resource with genetic, health, and lifestyle data for approximately 500,000 UK participants enrolled from 2006 to 2010 [7].

For drug efficacy analysis, we used drug prescriptions and laboratory measurements from primary care with records spanning roughly from 2000 to censoring in 2017. Biomarker responses to lipid-modifying drugs (ATC code C10) and antihypertensives (ATC codes C02-C09) were analyzed by comparing baseline and on-drug biomarker levels within a single drug class, excluding periods when other drugs for the same disease were prescribed. For the statin analysis, periods with concurrent use of fibrates or ezetimibe were excluded. Baseline measurements follow a drug-free period of ≥180 days and precede medication initiation by ≤730 days, while on-drug measurements occur 28–730 days post-initiation. The 28-day period marks the time for full therapeutic effect [8,9]. When multiple measurements exist within either period, we calculated linearly weighted averages that gave higher weight to measurements closer to the drug initiation. Prescription episodes were defined as continuous series of prescriptions for the same drug class, allowing ≤14 days gap between prescriptions. The coding for prescriptions and measurements are listed in https://github.com/fumi-github/drug_selection/tree/master/ukb/drugresponse

For drug choice analysis, medication data were obtained from nurse-led verbal interviews during the initial assessment (2006–2010, Data-Field 20003), and drug names were mapped to ATC codes[6]. For omega-3-triglycerides (C10AX06), only prescription Omacor 1g capsules were included, excluding dietary supplements.

Subjects were considered dyslipidemic if they had a diagnosis or self-reported dyslipidemia (ICD-10 code E78) by the initial assessment date, or if their LDL-C ≥190 mg/dL or HDL-C <40 mg/dL at initial assessment [10]. Similarly, subjects were classified as hypertensive if they had a diagnosis or self-reported hypertension (ICD-10 codes I10-I15) or recorded DBP ≥100 mmHg or SBP ≥160 mmHg [11].

Analyses were restricted to self-identified White British participants with consistent genetic ancestry [12], labeled as EUR (European descent), and participants of Indian, Pakistani, or Bangladeshi background by self-identification and genetic ancestry, labeled as SAS (South Asian descent). Genotypes were assayed using Affymetrix microarrays and imputed to the Haplotype Reference Consortium and UK10K reference panels (Data Category 100319) [12]. PGS for LDL-C and hypertension were obtained from Data-Field 26250 and 26244, respectively.

### 2.2. All of Us Research Program

The All of Us Research Program is a diverse cohort of approximately 630,000 USA participants enrolled since 2017 [13]. This study used data from the All of Us Research Program’s Controlled Tier Dataset v8, available to authorized users on the Research Workbench (workspace aou-rw-f61c46ea).

Drug prescription and laboratory measurement data were derived from electronic health records spanning roughly from 2010 to censoring in 2023. Drug efficacy analysis followed the same procedure as for UK Biobank, while drug choice was defined by prescription records extending beyond three months. Dyslipidemia was defined using the condition “Disorder of lipoprotein AND/OR lipid metabolism” (SNOMED code 48286001), and hypertension using “Hypertensive disorder” (SNOMED code 38341003). Analyses were restricted to participants with genetically predicted European descent. Genotyping was performed by short-read whole genome sequencing [14].

### 2.3. Variance-stabilizing Box-Cox transformation

The Box-Cox transformation is a family of power transformation that includes both the identity transformation (parameter λ = 1) and the logarithmic transformation (λ = 0) as special cases. We introduced variance-stabilizing Box-Cox transformation to equalize variances between baseline biomarker level *X* and on-drug level *Y*. The optimal transformation parameter was identified by minimizing the absolute value of the logarithm of the variance ratio, |log(Var [*Y*′]/Var [*X*′])|, for the transformed baseline and on-drug levels *X’* and *Y’*. The optimal parameter depends on the biomarker-drug combination and the cohort characteristics.

### 2.4. GWAS

To identify loci associated with drug response and drug choice, we conducted GWAS using Regenie software (version 3.4.1.gz) [15]. The analysis involved two steps: computing per-chromosome genetic predictions for SNPs directly genotyped on microarrays or a subset of mutually independent variants (for All of Us, plink2 --indep-pairwise 200kb 1 0.5), followed by association testing with all SNPs. In Step 1, SNPs were excluded if they had minor allele frequency (MAF) <0.01, minor allele count <100, missing call rate >0.1, or Hardy-Weinberg equilibrium P-value <10^−15^. Subjects with missing call rates >0.1 were removed. In Step 2, multiallelic SNPs and those with MAF <0.01 were excluded. Additional filters removed imputed SNPs with imputation accuracy ≤0.4, and sequenced SNPs with missing call rate >0.1 or Hardy-Weinberg deviation beyond ±20%. For binary traits, Firth likelihood ratio tests were applied to variants with P-value <0.01.

For the drug choice analysis, covariates included sex, age, BMI, age^2^, sex-by-age interaction, and genetic principal components (top 20 for UK Biobank and top 16 for All of Us). The resulting linkage disequilibrium (LD) score regression intercepts (0.999–1.039 for dyslipidemia drug choice and 1.018–1.036 for hypertension drug choice) indicate absence of population stratification.

For the drug response analysis, to avoid improper adjustment of the composite phenotype (the difference between baseline and on-drug biomarker levels), only genetic principal components were used as covariates. GWAS summary statistics from UK Biobank and All of Us were combined via meta-analysis using the METAL software (version 2020–05-05) [16], limited to SNPs present in both datasets. The resulting LD score regression intercepts (0.989–0.994 for LDL-C response and 0.897–0.994 for SBP response) likewise indicate negligible population stratification. Manhattan plots were drawn using the qqman package (version 0.1.9) [17] of the R software (version 4.4.0) [https://cran.r-project.org/].

Genome-wide significance was defined as P-value <5×10^−8^. Significant SNPs were compared against previous GWAS results using the hugeamp database [https://hugeamp.org/] and the GWAS ATLAS [https://atlas.ctglab.nl/PheWAS].

Our drug choice GWAS was compared with the drug use GWAS from FinnGen [18], in which cases were defined as individuals who purchased the drug and controls as all other participants in the cohort [https://r12.finngen.fi/pheno/RX_STATIN https://risteys.finngen.fi/endpoints/RX_STATIN https://r12.finngen.fi/pheno/RX_ANTIHYP https://risteys.finngen.fi/endpoints/RX_ANTIHYP].

### 2.5. Heritability and genetic correlation

Because drug choice is a binary trait (prescribed vs. not prescribed) defined among patients with a given disease, we estimated heritability specific to the patient subgroup, quantifying the proportion of drug choice variance explained by SNPs. Heritability and genetic correlations were estimated using LD score regression (LDSC software version 1.0.1) [19], which was previously applied in a sex-specific heritability study [20]. Input data included GWAS summary statistics recomputed using PLINK software (version 2.00a2.3LM) [https://www.cog-genomics.org/plink/2.0/] under similar quality control criteria and covariates (excluding the sex-by-age interaction term), published GWAS summary statistics, and the HapMap3 European dataset as reference. Correlation matrices were visualized using the corrplot package (version 0.94) of the R software.

### 2.6. Development of drug choice PGS

To predict drug choice traits, we developed PGS by combining index SNPs from external GWAS for relevant disease traits and fitting weights. We took this approach of weighting the index SNPs, rather than developing PGS from scratch based on drug choice GWAS, because of the limited sample size of the GWAS and to leverage the genetic correlation between drug choice traits and disease traits. An individual’s PGS was calculated as a weighted sum of allele-dose genotypes (coded 0, 1, or 2). To ensure proper validation, index SNPs were taken from disease GWAS that excluded UK Biobank and All of Us, namely studies on lipids [21], blood pressure [22–24], type 2 diabetes [25], BMI [26], and CAD [27]. After excluding SNPs with MAF <0.01 or located on sex chromosomes, 570 SNPs remained.

A basic score was defined as a weighted sum of sex, age, and BMI, while a combined score summed the basic score and PGS. Development of these scores was performed in R using the glmnet package (version 4.1–7). The UK Biobank EUR subjects were split into 80% for model training and tuning and 20% for blind testing (in 20 times repeated independent sub-sampling). Within the 80% subset, data was further divided into 60% for training and 20% for tuning (repeated 20 times) to fine-tune the Lasso penalty hyperparameter.

On the training set, we first performed logistic regression with drug choice as the dependent variable and demographic factors (sex, age, and BMI) as independent variables, including 20 genetic principal components as covariates. The resulting coefficients defined the weights of the basic score. Next, we took the residuals from this regression and applied Lasso regressions (penalty chosen from 0.001 to 1) with SNPs as independent variables. The resulting coefficients defined the weights of a tentative PGS. The variable selection functionality of Lasso regression assigns zero weights to SNPs in LD with other SNPs that show stronger association.

On the tuning set, we performed logistic regressions with drug choice as the dependent variable and the tentative PGS (trained under various penalties) as the independent variable, and selected the Lasso penalty yielding the highest Z score as the optimal hyperparameter. Finally, to scale both scores accurately, we ran another logistic regression incorporating the basic score and the tentative PGS as independent variables. The coefficient for the basic score was approximately one because we are essentially replicating the logistic regression computed on the training set, indicating that no further rescaling was needed. By contrast, the coefficient for the tentative PGS, this time in an unpenalized regression and concurrently modeled with the basic score which is a stronger predictor, could differ from one and was defined as the scaling factor. We obtained the final PGS by multiplying the tentative PGS weights by this scaling factor. As a result, both the basic score and the final PGS were scaled to have a unit effect size on the log-odds of drug choice.

### 2.7. Validation of drug choice PGS

Internal validation used the reserved 20% of UK Biobank EUR subjects. External validation was performed using the All of Us cohort and the UK Biobank SAS. Predictive performance was evaluated using the Area Under the Receiver Operating Characteristic Curve (AUC-ROC) analyses for three models: PGS alone, basic score alone, and their sum. Additionally, we evaluated prediction accuracy by analyzing drug choice frequency across score deciles for each model.

For external validation, the final SNP weights were derived by averaging across all 20 cross-validation runs in the UK Biobank EUR. SNP weight signs were corrected for differences in genome builds (GRCh37 in UK Biobank and GRCh38 in All of Us) using the triple-liftOver program (version 1.33) [28].

## 3. Results

### 3.1. GWAS for drug efficacy

We analyzed LDL-C response to hypolipidemic drugs and systolic blood pressure (SBP) response to antihypertensive drugs using data from the UK Biobank and the All of Us Research Program (Table 1). We performed GWAS for medication-biomarker combinations with >1000 observations in both cohorts. For those combinations, on-drug biomarker levels were significantly lower than baseline levels in all except for SBP response to calcium channel blockers in All of Us.

**Figure 1:**
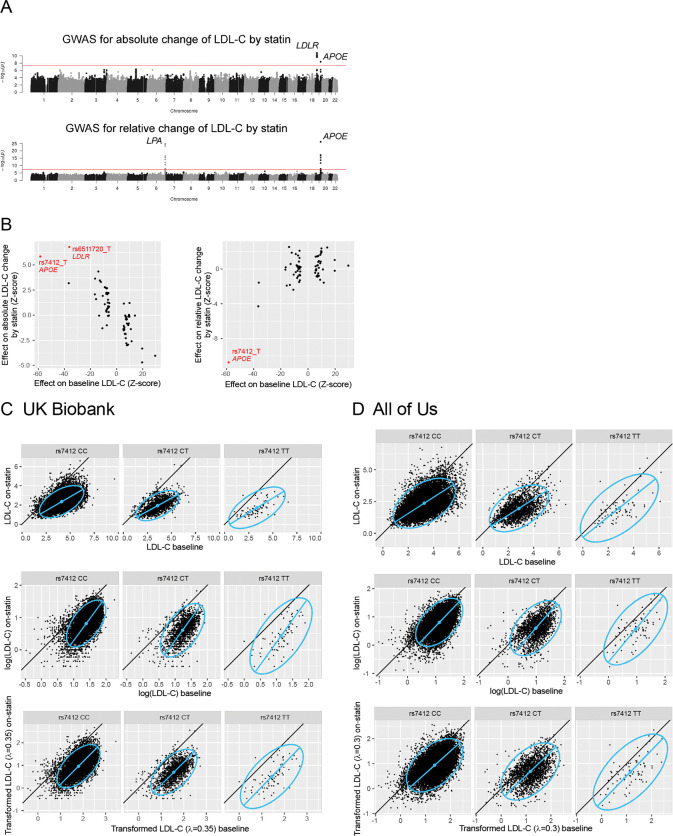
GWAS for LDL-C response on statin. **A**. Manhattan plots of GWAS for LDL-C response to statin based on two phenotype definitions: absolute LDL-C change (top) and relative change (bottom). Variants are plotted as points, with chromosomal position along the horizontal axis and −log_10_(P-value) along the vertical axis. The red line marks the genome-wide significant level of P = 5×10^−8^. **B**. For 71 genetic loci previously associated with baseline LDL-C, the effects of index SNPs on baseline LDL-C (horizontal axis) are compared with their effects on absolute LDL-C change by statin (vertical axis of left panel) and relative change (vertical axis of right panel). SNPs highlighted in red reached genome-wide significance for drug response. In UK Biobank (**C**) and All of Us (**D**), the baseline LDL-C levels of participants (horizontal axis) are compared with the on-statin levels (vertical axis), where LDL-C is scaled in raw scale (top panels), log-scale (middle panels) and variance-stabilizing Box-Cox scale (bottom panels). Points represent individual participants, separated by genotypes of SNP rs7412. Blue ellipses indicate the 95-percentile contours of a fitted bivariate normal distributions, and the blue line segments shows the first principal component, visualizing the data distribution. Black diagonal lines mark equality between the two axes.

Several variants have previously been reported as genetic predictors of LDL-C response to statins [1,2]. We quantified LDL-C response using two conventional metrics: absolute change (on-drug level *Y* minus baseline level *X*) and relative change (log(*Y*/*X*)). When combining results from the UK Biobank and All of Us, we identified genome-wide significant associations at the *APOE*, *LDLR* and *LPA* loci, consistent with previous reports (Figure 1A).

The *APOE* locus demonstrated associations with both absolute and relative LDL-C change in response to statin therapy. However, we observed a paradoxical pattern where the T allele of SNP rs7412, which encodes the *APOE* E2 isoform and is associated with reduced baseline LDL-C, exhibited a positive effect on absolute LDL-C change (beta = 0.080, P = 4.8×10^−9^) but a negative effect on relative change (beta = −0.051, P = 6.6×10^−27^). This contradictory pattern was not due to analytical error, as similar results were observed in a separate UK Biobank study [29], where the same allele a showed positive effect on absolute change (beta = 0.053, P = 0.011) and a negative effect on relative change (beta = −0.232, P = 1.1×10^−28^).

To assess whether this pattern generalizes, we systematically examined 71 genetic loci previously associated with baseline LDL-C in an independent GWAS [21] that did not include UK Biobank or All of Us participants. Variants’ effects on baseline LDL-C were systematically opposite to their effects on absolute LDL-C change (Figure 1B, left panel) but aligned with their effects on relative LDL-C change (Figure 1B, right panel). This consistent inversion pattern suggests a methodological artifact rather than genuine biological effects on drug response.

### 3.2. Scaling of biomarker levels

To explore the mechanism underlying this consistent inversion, we examined the joint distribution of baseline and on-statin LDL-C values. In UK Biobank, on-statin LDL-C levels were consistently lower than baseline levels, but the magnitude of reduction was smaller in subjects with lower baseline LDL-C (Figure 1C, top panel). This relationship is illustrated by the distribution-fitting blue line with a slope less than 45 degrees, which was consistent across different genotypes of rs7412. Subjects with the TT genotype, who have lower baseline LDL-C, experienced less absolute decreases, explaining the positive association between rs7412_T and absolute LDL-C change.

For relative change analysis, we examined the distribution after logarithmic transformation of both baseline and on-statin LDL-C values (Figure 1C, middle panel). Under log-scale, subjects with lower baseline LDL-C showed proportionally larger reductions, resulting in subjects with TT genotype experiencing greater relative decreases, which explains the negative association between rs7412_T and relative LDL-C change. The same patterns were observed in All of Us data (Figure 1D, top and middle panels).

This phenomenon arises from unequal variance between baseline and on-statin LDL-C distributions. Pharmacogenetic studies of drug efficacy aim to identify genetic variants influencing the change from baseline (*X*) to on-drug (*Y*) levels while excluding variants associated solely with either *X* or *Y*. However, this goal is compromised when the change measure (*Y* – *X*) is strongly correlated with *X* or *Y*. Under raw scaling (absolute change), the distribution is elongated horizontally, with greater variance in *X* than in *Y*, producing a strong correlation between *Y* − *X* (≈ −*X*) and *X* (r = −0.70 in UK Biobank; Figure 1C, top panel). Under logarithmic transformation (relative change), the distribution elongates vertically with greater variance in *Y* than *X*, yielding a strong correlation between *Y* − *X* (≈ *Y*) and *Y* (r = 0.60 in UK Biobank; Figure 1C, middle panel).

Both raw scaling and log-scaling are special cases of the Box-Cox family of transformations with parameters λ = 1 and 0, respectively. To address these artifacts, we applied a variance-stabilizing Box-Cox transformation to equalize the variances between baseline and on-drug biomarker levels. Under this transformation, the distribution axis approaches a 45-degree angle, minimizing spurious correlations. The optimal Box-Cox parameters were λ = 0.35 for UK Biobank (Figure 1C, bottom panel) and λ = 0.3 for All of Us (Figure 1D, bottom panel). The optimal λ values for SBP response on diuretic, β-blocker, calcium channel blocker and RAS-acting agents were 2.00, −1.15, −1.15, −0.20 in UK Biobank and 1.85, 2.00, −0.75, −0.70 in All of Us. In situation of baseline biomarker level being higher than the on-drug level, the optimal λ becomes high when the baseline standard deviation is smaller than the on-drug standard deviation (as for diuretic, see Table 1B), and low when opposite (as for β-blocker, calcium channel blocker in UK Biobank and RAS-acting agents in both cohorts). Importantly, when we performed GWAS using these variance-stabilized measures, no genome-wide significant associations were detected for LDL-C response to hypolipidemics or SBP response to antihypertensives in the combined analysis of UK Biobank and All of Us (Supplementary Figure 1).

### 3.3. GWAS for drug choice

We next explored the genetic architecture underlying drug choice among patients with dyslipidemia or hypertension by comparing those prescribed a particular drug with those who were not. In UK Biobank European descent (EUR) subjects with dyslipidemia (Table 2A), GWAS on drug choice identified multiple significant loci: 12 for statin, 3 for fibrate, and 2 for ezetimibe (Table 3). These loci overlapped with previously reported associations for lipid traits as well as blood pressure, coronary artery disease (CAD), and type 2 diabetes (T2D). Comparison with statin *use* GWAS in FinnGen [18] showed replication for 13 of 14 SNPs (93%) at genome-wide significance.

Among UK Biobank EUR hypertensive subjects (Table 2B), we identified genome-wide significant loci for drug choice: 6 for diuretic, 3 for β-blocker, 8 for calcium channel blocker, and 6 for RAS-acting agents (Table 4). These loci also overlapped with those associated with blood pressure, lipid levels and CAD, indicating shared genetic architecture across cardiometabolic traits. Comparison with antihypertensive *use* GWAS in FinnGen showed replication for 16 of 18 SNPs (89%) at genome-wide significance.

### 3.4. Genetic correlations between drug choice and disease traits

**Figure 2:**
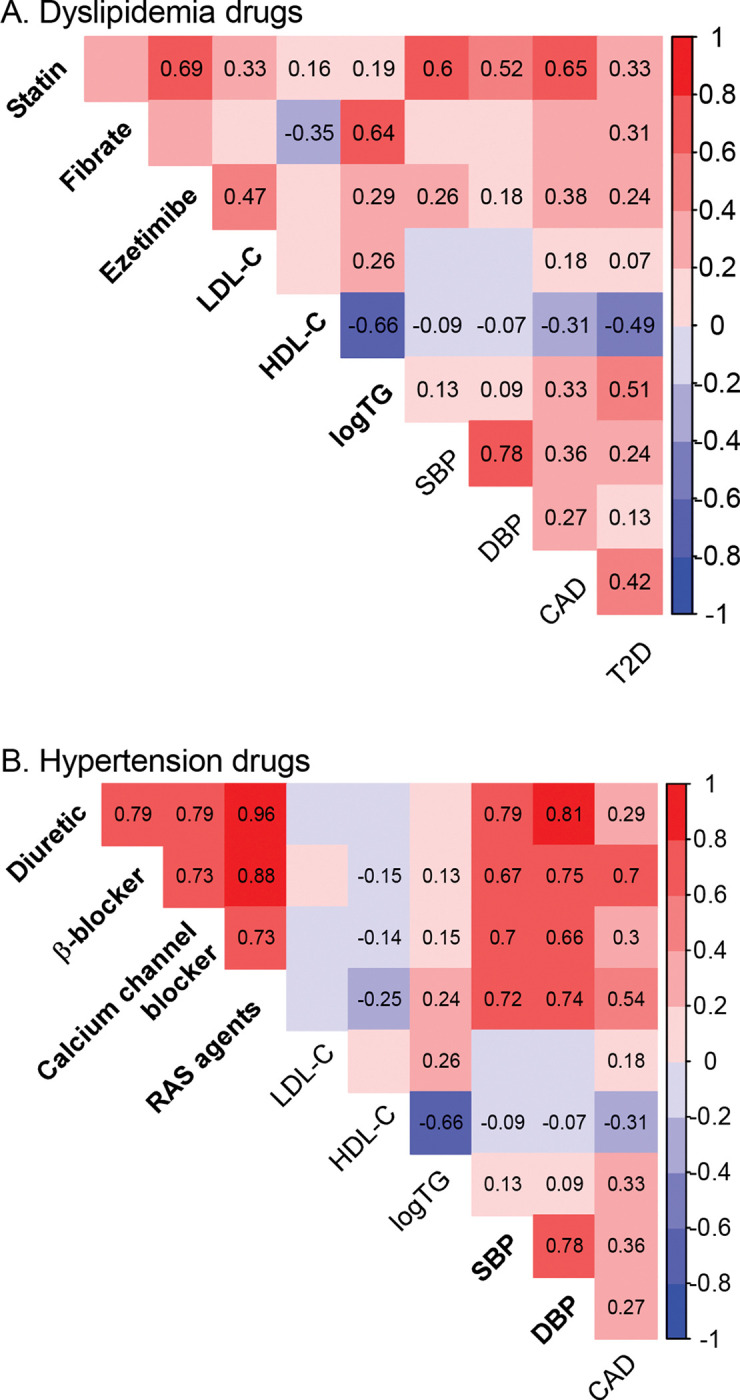
Genetic correlation analysis. Heatmaps shows genetic correlations between drug choice traits and cardiometabolic disease traits for dyslipidemia (**A**) and hypertension (**B**) medications. Disease traits include LDL-C, HDL-C, triglyceride levels (logTG), systolic blood pressure (SBP), diastolic blood pressure (DBP), coronary artery disease (CAD) and type 2 diabetes (T2D). Color intensity indicates correlation magnitude, and numerical values are displayed for significant correlations (P-value <0.01). Drug names and their primary target disease traits are highlighted in bold font.

To characterize the shared genetic basis between drug choice and cardiometabolic diseases, we estimated SNP heritability and genetic correlations (Figure 2). Among dyslipidemia medications, statin showed the highest heritability (0.054, SE = 0.005), followed by ezetimibe (0.011, SE = 0.003) and fibrate (0.008, SE = 0.004). Statin choice correlated most strongly with blood pressure and CAD, fibrate choice with HDL-C (negative direction) and triglycerides, and ezetimibe choice with LDL-C. All three medications showed moderate correlations with T2D.

For hypertension medications, RAS-acting agents had the highest heritability (0.033, SE = 0.004), followed by calcium channel blocker (0.030, SE = 0.004), diuretic (0.019, SE = 0.004), and β-blocker (0.017, SE = 0.003) for. All antihypertensives displayed mutual genetic correlations and significant correlations with blood pressure. β-blocker choice correlated most strongly with CAD, while RAS-acting agents correlated most strongly with HDL-C (negative direction) and triglycerides.

### 3.5. PGS for drug choice

We developed PGS to predict drug choice, leveraging the observed genetic overlap between drug choice and cardiometabolic traits (Tables 3, 4, and Figure 2). A total of 570 SNPs associated with relevant traits from published literature were compiled and weighted to generate predictive models. The model development utilized 80% of UK Biobank EUR subjects, with validation performed on the remaining UK Biobank subjects, the All of Us cohort, and the UK Biobank South Asian descent (SAS) subjects (Table 2). Alongside the PGS, we derived a basic score based on sex, age and BMI, as well as a combined score by summing the basic score and PGS.

For dyslipidemia drugs, validation in UK Biobank EUR showed varying predictive performance measured by the Area Under the Receiver Operating Characteristic Curve (AUC-ROC): 0.550–0.621 for the PGS, 0.619–0.694 for the basic score, and 0.633–0.702 for the combined score (Table 5A). Although the combined score demonstrated a statistically significant improvement over the basic score, the increase was modest (0.008–0.056). In the All of Us cohort, PGS showed modest yet statistically significant performance. The AUC-ROC for PGS was similar or larger in UK Biobank SAS subjects than in the EUR subjects, even though the scores were trained in EUR subjects. Among the three drugs, fibrate achieved the highest prediction performance across all cohorts.

**Figure 3:**
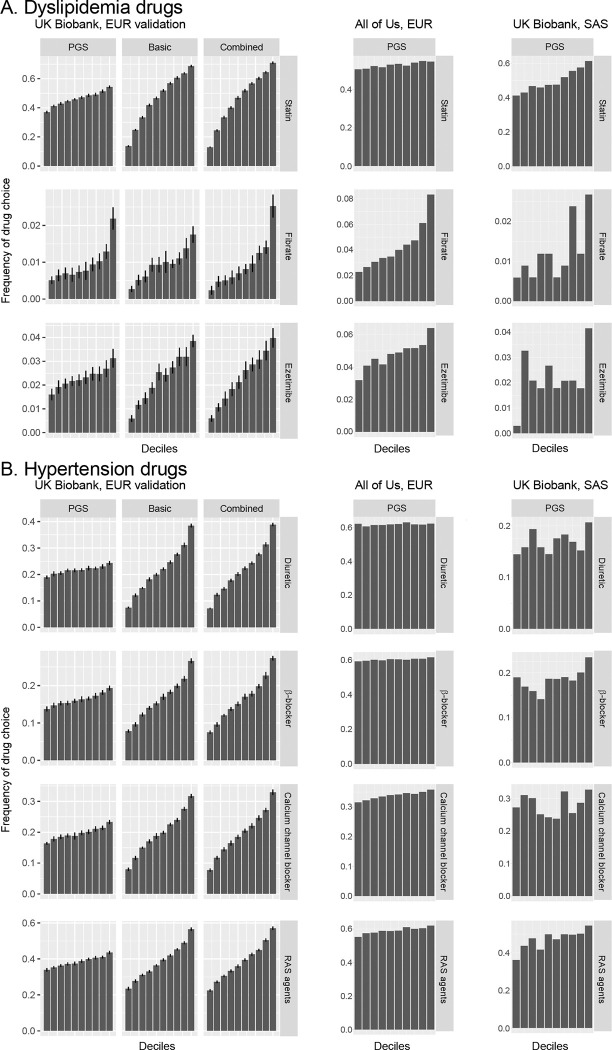
Drug choice frequencies across PGS deciles. This figure compares drug choice frequencies across score deciles for dyslipidemia (**A**) and hypertension (**B**) medications in the UK Biobank EUR validation cohort (left panels), the All of Us cohort (middle panels), and UK Biobank SAS (right panels). Each row corresponds to a specific drug, and columns represent different prediction score types (PGS, basic score, and combined score). Bar heights indicate the proportion of subjects prescribed the drug within a given decile. In UK Biobank EUR, error bars represent the standard deviation across 20 cross-validation trials. EUR, European descent; SAS, South Asian descent.

Analysis of drug choice frequencies by score deciles in UK Biobank EUR (Figure 3A) revealed clear gradients across both the PGS and basic score, with the combined score showing the greatest separation. In the top decile for the combined statin score, sensitivity—the proportion of individuals prescribed the drug—was 71%. Similar patterns were observed in the All of Us cohort and UK Biobank SAS, though gradients in UK Biobank SAS were less uniform due to the smaller sample size. Notably, drug-specific PGS were more predictive for their intended drugs than PGS for other medications, confirming their specificity (Supplementary Figure 2A). Statin prescriptions increased with higher statin PGS (first row) and a similar trend was observed for fibrate (second row). However, ezetimibe PGS did not outperform other PGS in predicting ezetimibe prescription.

For hypertension drugs, overall predictive performance was lower. In UK Biobank EUR, AUC-ROC values were 0.524–0.534 for PGS, 0.614–0.649 for the basic score, and 0.620–0.651 for the combined score (Table 5B). Although the improvements from adding PGS to the basic score were statistically significant, they remained small (0.002–0.007). In the All of Us cohort, the PGS achieved statistical significance for all antihypertensives except diuretic. In UK Biobank SAS, AUC-ROC values were comparable to those of UK Biobank EUR.

Decile analysis in UK Biobank EUR demonstrated modest PGS gradients for hypertension drugs but more pronounced gradients for the basic score, with marginal gains from combining the two (Figure 3B). For the top decile of combined score for RAS-acting agents, the sensitivity was 57%. In the All of Us cohort and UK Biobank SAS, PGS showed the clearest separation for RAS-acting agents. Drug-specific PGS demonstrated stronger predictive power for their intended medications than PGS for other medications, although this specificity was less pronounced than for dyslipidemia drugs (Supplementary Figure 2B).

## 4. Discussion

We investigated whether germline genetic variation influences drug response and drug choice for dyslipidemia and hypertension, using data from the UK Biobank and All of Us cohorts. Although prior studies reported SNPs modifying LDL-C response to statins, we found that these associations disappeared once the biomarker scaling issue was addressed. In contrast, GWAS for drug choice identified several significant loci overlapping with established cardiometabolic disease loci. Genetic correlation analysis revealed distinct patterns of genetic sharing between drug choice and disease traits. Building on these observations, we developed drug choice PGS using SNPs associated with relevant cardiometabolic traits. The PGS demonstrated modest yet statistically significant predictive performance, especially for dyslipidemia medication choice (AUC-ROC 0.550–0.621 in UK Biobank). When combined with demographic factors (age, sex and BMI), predictive performance increased further (AUC-ROC 0.633–0.702). External validation in the All of Us cohort confirmed the generalizability of these findings, although with slightly attenuated performances. Notably, the PGS developed in UK Biobank EUR performed comparably in UK Biobank SAS, suggesting cross-ancestry transferability.

### 4.1. Spurious drug response associations caused by biomarker scaling

Pharmacogenetic studies typically quantify drug-induced biomarker response as either absolute change (on-drug level *Y* minus baseline level *X*) or relative change, computed as *Y*/*X* – 1 or log(*Y*/*X*), which are mutually convertible and yield similar values. Previous GWAS of LDL-C response to statins have used measures of absolute change [29,30] or relative change [2,29–32].

Our analysis revealed that genetic variants associated with baseline LDL-C display systematically opposite associations with absolute LDL-C change (Figure 1B, left panel) but concordant associations with relative change (Figure 1B, right panel) in response to statin therapy. Absolute change represents the difference between raw LDL-C values (*Y* – *X*), while relative change represents the difference between log-transformed values (log(*Y*) – log(*X*)). After applying a variance-stabilizing Box-Cox transformation, which represents an intermediate scaling between identity and logarithmic transformations and equalizes drug effects across the baseline biomarker range (Figure 1C bottom panel, 1D bottom panel), no variants remained significantly associated with LDL-C response to statins.

Previous studies recognized that variants associated with baseline LDL-C can spuriously appear associated with LDL-C change, leading some researchers to adopt baseline-adjusted residuals as the target phenotype [33]. However, introduction of false associations by baseline adjustment has been demonstrated through analytical derivations [29,34], simulation studies [35], and empirical analyses[29,32]. A previous study[33] employed the Box-Cox transformation with a different objective, to ensure that the residuals from the regression of on-drug LDL-C level on baseline LDL-C fits a Gaussian distribution. Here the optimal parameters for power transformation were λ = 0.16, 0.48 and 0.68 in three cohorts, values lying between the identity and logarithmic transformations.

To our knowledge, this study is the first to demonstrate that biomarker scaling methods can systematically generate spurious pharmacogenetic signals and to propose the variance-stabilizing Box-Cox transformation as a solution. This methodological insight has broad implications for pharmacogenomic research, suggesting that some previously reported genetic associations with drug response may be artifacts arising from skewed data distributions—particularly when the optimal Box-Cox parameter substantially differs from the identity (λ = 1) or logarithmic (λ = 0) cases. Future pharmacogenetic studies should consider applying variance-stabilizing transformations to mitigate these artifacts and to ensure more reliable identification of genuine genetic predictors of drug response.

### 4.2. Genetic determinants of drug choice trait

This study introduces the concept of genetic determinants of the “drug choice trait”, demonstrated through development of PGS for dyslipidemia and hypertension medication choices. Our approach uniquely defines cases as patients prescribed a given drug and controls as those with the same disease who were not prescribed that drug. This differs from previous studies on the “drug use trait,” which defined controls as all non-drug users, including healthy individuals [6]. In the context of personalized medicine, the former trait captures which drug to pick at the time of diagnosis, and the latter trait captures the drug to pick regardless of disease onset. For example, statin drug choice in UK Biobank and statin drug use in FinnGen [18] showed a moderate genetic correlation of 0.644 (SE: 0.005).

The genetic architecture of drug choice traits showed varying degrees of overlap with cardiometabolic disease traits (Figure 2), consistent with known pharmacological mechanisms. Among dyslipidemia medications, fibrate choice correlated most strongly with triglycerides, consistent with its PPARα-activation mechanism and effects on fatty acid catabolism. Ezetimibe choice correlated primarily with LDL-C, reflecting its clinical role as an add-on therapy when statin monotherapy is insufficient. Statin choice correlated most strongly with blood pressure and CAD, aligning with its widespread use in cardiovascular risk management. For hypertension medications, β-blocker choice demonstrated the strongest correlation with CAD, in line with its mechanism of reducing catecholamine effects and heart rate. The correlation between RAS-acting agent choice and triglycerides corresponds with RAS’s known influence on hepatic and skeletal muscle fatty acid metabolism [36].

### 4.3. Limited utility of drug choice PGS

Although germline genetics may eventually guide medication choice, our current PGS for hypolipidemic and antihypertensive drug choice offers limited predictive utility. In this study, individuals in the top combined-score decile exhibited 71% sensitivity for statin and 57% for RAS-acting agent prescriptions, but lower sensitivity for other drug classes (Figure 3).

Our PGS methodology builds upon the concept of partitioned PGS for diabetes [37,38], but instead of partitioning SNPs into subgroups, we weighted cardiometabolic disease SNPs based on their association with drug choice traits. This approach differs from previous pharmacogenomic PGS studies, which focused on predicting drug outcomes (toxicity, efficacy, or optimal dosage) either through small drug trial cohorts [39] or by leveraging disease-related PGS [40]. Appropriation of disease-related PGS for drug choice prediction showed variable performance across different drug classes in our analyses. For the antihypertensives, which exhibit high genetic correlation with blood pressure (Figure 2B), the drug choice traits could be predicted by the PGS for hypertension (AUC-ROC 0.545–0.561) slightly more accurately than by the PGS’s trained for the respective drugs. In contrast, ezetimibe, statin and fibrate exhibit moderate to no genetic correlation with LDL-C (Figure 2A), and the LDL-based PGS showed performance ranging from aligned to inverse direction (AUC-ROC 0.584, 0.517 and 0.487, respectively).

Drug suitability can be conceptualized as the composite of safety, efficacy, and pathological appropriateness. Simplistically, interindividual variability in drug suitability can be regarded as a combination of interindividual variability in the three factors. For cardiometabolic disease drugs, the variability in safety is likely minimal because widely used drugs generally lack severe toxicity in subpopulations. In terms of efficacy, known genetic variants affecting drug efficacy reportedly show relatively small effects (e.g., ~1% effect on LDL-C by variants in *LPA*, *APOE*, *SORT1*, *SLCO1B1* for statins, and odds ratio of 1.25 for metformin response in *ATM* variants) [1] although our analysis suggests some reported effects may reflect methodological artifacts. Consequently, genetic difference in drug suitability may predominantly arise from interindividual variability in pathological appropriateness.

Although our drug choice PGS exhibited statistically significant predictability, the modest AUC-ROC values indicate room for improvement. Enhancements could stem from expanding the SNP set and refining allele weighting. Our current PGS includes 570 SNPs derived from cardiometabolic disease GWAS published until 2017, deliberately excluding UK Biobank and All of Us data to ensure rigorous validation. Incorporating more recent large-scale GWAS findings and anticipated studies of rare variants with greater effect sizes would expand the SNP set. More importantly, refining SNP allele weighting with data from large genomic-prescription cohorts is necessary to increase accuracy.

While drug choice PGS could eventually assist in clinical medication matching, several limitations remain. First, the PGS captures real-world prescribing behavior rather than direct pharmacodynamic or outcome-based efficacy. A higher PGS does not necessarily imply a superior therapeutic response, which can only be evaluated through randomized clinical trials. Although clinical trial-based pharmacogenomic studies enable precise assessment of drug dose and administration, strict trial inclusion criteria may introduce selection bias. Second, we did not analyze how cross-cohort difference in clinical guidelines, such as those between the NICE versus American recommendations and their temporal versions, influence medication choice in UK Biobank and All of Us. Third, prescribed medications in the training data may not always represent optimal patient-drug matches. The modest predictive performance of genetics alone underscores the need for integrated models combining genetic, demographic and clinical features to achieve more practical drug recommendation systems.

## Supplementary Material

Supplement 1

## Figures and Tables

**Table 1A.** Characteristics of subjects analyzed for hypolipidemic response

	UK Biobank, EUR		All of Us, EUR	
		
Total subjects	15,426		14,894	
Sex male (N, %)	8,518	55%	7,488	51%
Age (mean, SD)	61.3	6.0	69.4	11.0
BMI (mean, SD)	28.7	4.8	30.2	6.3
C10AA, Statin				
Drug response observed (N)	15,217		13,775	
LDL-C baseline [mmol/L] (mean, SD)	4.04	0.96	3.20	1.01
LDL-C on-drug [mmol/L] (mean, SD)	2.30	0.69 *	2.32	0.81 *
C10AB, Fibrate				
Drug response observed (N)	78		404	
LDL-C baseline [mmol/L] (mean, SD)	4.20	1.11	2.75	0.88
LDL-C on-drug [mmol/L] (mean, SD)	3.56	0.92 *	2.89	0.93
C10AC, Bile acid sequestrants				
Drug response observed (N)	28		180	
LDL-C baseline [mmol/L] (mean, SD)	3.44	1.12	2.95	0.95
LDL-C on-drug [mmol/L] (mean, SD)	3.05	1.12	2.81	0.90
C10AD, Nicotinic acid and derivatives				
Drug response observed (N)	4		313	
LDL-C baseline [mmol/L] (mean, SD)	4.79	0.63	3.07	0.91
LDL-C on-drug [mmol/L] (mean, SD)	4.38	0.62	3.02	0.94
C10AX06, Omega-3-triglycerides				
Drug response observed (N)	23		258	
LDL-C baseline [mmol/L] (mean, SD)	3.15	1.06	3.03	0.84
LDL-C on-drug [mmol/L] (mean, SD)	3.45	0.96	3.10	0.85
C10AX09, Ezetimibe				
Drug response observed (N)	151		273	
LDL-C baseline [mmol/L] (mean, SD)	4.16	0.93	3.61	1.08
LDL-C on-drug [mmol/L] (mean, SD)	3.27	0.81 *	3.02	0.97 *

**Table 1B.** Characteristics of subjects analyze for antihypertensive response

	UK Biobank, EUR		All of Us, EUR	
		
Total subjects	28,189		26,926	
Sex male (N, %)	14,276	51%	10,482	39%
Age (mean, SD)	59.8	6.9	63.2	15.1
BMI (mean, SD)	28.9	5.0	30.0	6.8
C02, Various antihypertensives				
Drug response observed (N)	-		2,300	
SBP baseline [mmHg] (mean, SD)	-	-	125.0	13.2
SBP on-drug [mmHg] (mean, SD)	-	-	124.8	13.7
C03, Diuretic				
Drug response observed (N)	5,269		6,230	
SBP baseline [mmHg] (mean, SD)	158.3	13.8	123.9	13.1
SBP on-drug [mmHg] (mean, SD)	146.3	14.6 *	123.1	13.8 *
C07, β-blocker				
Drug response observed (N)	5,913		11,403	
SBP baseline [mmHg] (mean, SD)	144.8	19.1	125.9	13.7
SBP on-drug [mmHg] (mean, SD)	135.2	16.8 *	124.8	13.8 *
C08, Calcium channel blocker				
Drug response observed (N)	7,428		1,858	
SBP baseline [mmHg] (mean, SD)	156.4	15.6	124.3	12.4
SBP on-drug [mmHg] (mean, SD)	141.3	13.3 *	124.6	13.0
C09, RAS-acting agents				
Drug response observed (N)	10,723		7,498	
SBP baseline [mmHg] (mean, SD)	154.0	15.6	135.9	14.4
SBP on-drug [mmHg] (mean, SD)	140.6	14.5 *	131.1	13.5 *

ATC codes of drugs are indicated. EUR, European descent

* On-drug biomarker level is lower than baseline (P < 0.05).

**Table 2A.** Characteristics and drug usage of the dyslipidemia cohort

	UK Biobank, EUR		All of Us, EUR		UK Biobank, SAS	
			
Total subjects	116,305		87,006		3,367	
Sex male (N, %)	69,587	60%	39,274	46%	2,269	67%
Age (mean, SD)	59.0	7.4	63.9	12.9	54.7	8.5
BMI (mean, SD)	28.9	4.6	30.2	6.6	28.0	4.2
Diabetes (N, %)	7,535	6%	25,997	30%	584	17%
Hypertension (N, %)	58,323	50%	61,159	70%	1,580	47%
Ischemic heart diseases (N, %)	15,658	13%	16,615	19%	592	18%
Drug usage (N, %)						
C10AA, Statin	53,671	46%	45,875	53%	1,679	50%
C10AB, Fibrate	1,113	1%	3,697	4%	41	1%
C10AC, Bile acid sequestrants	97	0%	1,916	2%	0	0%
C10AD, Nicotinic acid and derivatives	74	0%	2,856	3%	3	0%
C10AX06, Omega-3-triglycerides	404	0%	2,844	3%	21	1%
C10AX09, Ezetimibe	2,666	2%	4,162	5%	74	2%

**Table 2B.** Characteristics and drug usage of the hypertension cohort

	UK Biobank, EUR		All of Us, EUR		UK Biobank, SAS	
			
Total subjects	137,348		80,447		2,896	
Sex male (N, %)	73,035	53%	36,892	46%	1,695	59%
Age (mean, SD)	60.0	6.9	63.5	13.7	57.2	7.9
BMI (mean, SD)	29.0	4.8	31.0	6.9	28.4	4.5
Diabetes (N, %)	8,159	6%	26,255	33%	564	19%
Dyslipidaemia (N, %)	58,323	42%	61,159	76%	1,580	55%
Ischemic heart diseases (N, %)	14,625	11%	16,555	21%	571	20%
Drug usage (N, %)						
C02, Various antihypertensives	5,249	4%	13,370	17%	126	4%
C03, Diuretic	29,764	22%	49,822	62%	489	17%
C07, β-blocker	22,369	16%	48,476	60%	533	18%
C08, Calcium channel blocker	27,049	20%	27,230	34%	813	28%
C09, RAS-acting agents	52,788	38%	47,589	59%	1,363	47%

ATC codes of drugs are indicated. EUR, European descent; SAS, South Asian descent

**Table 3.** Significant loci for GWAS of dyslipidemia drug choice

							Statin		Fibrate		Ezetimibe		FinnGen*	
Chr	Position_hg19	Nearby gene	SNP ID	Effect allele	Other allele	Effect allele frequency	log_OR	P	log_OR	P	log_OR	P	P	Other associated traits**
					
1	55,505,647	*PCSK9*	rs11591147	G	T	0.99	**0.239**	**4.9E-09**	−0.147	4.3E-01	0.731	1.6E-06	**0**	LDL
1	109,818,306	*PSRC1*	rs629301	G	T	0.20	**−0.063**	**3.0E-08**	−0.101	6.0E-02	−0.182	5.5E-07	**3.0E-174**	LDL
2	21,266,774	*APOB*	2:21266774_	GGCAGCGCCA	G	0.64	−0.026	6.6E-03	−0.046	3.1E-01	**−0.170**	**3.5E-09**	NA	LDL
2	27,730,940	*GCKR*	rs1260326	T	C	0.40	**0.051**	**2.6E-08**	0.220	3.0E-07	0.078	6.4E-03	**6.2E-36**	TG
6	161,005,610	*LPA*	rs55730499	C	T	0.91	**−0.104**	**5.4E-11**	−0.040	6.0E-01	**−0.332**	**1.7E-13**	**8.1E-82**	Lipoprotein(a), LDL
6	161,089,307	*LPA*	rs56393506	C	T	0.82	**−0.089**	**1.1E-13**	0.076	1.9E-01	−0.172	1.8E-06	**4.3E-53**	Lipoprotein(a), LDL
8	9,184,231	*RP11–10A14.4*	rs4240624	G	A	0.09	**−0.097**	**6.8E-10**	−0.010	8.9E-01	−0.042	3.9E-01	**1.2E-26**	HDL
9	22,103,813	*CDKN2B-AS1*	rs1333042	A	G	0.50	**−0.081**	**6.1E-19**	−0.075	8.4E-02	−0.050	7.8E-02	**3.3E-42**	CAD
9	107,661,742	*ABCA1*	rs2740488	A	C	0.74	**0.073**	**1.7E-12**	0.036	4.6E-01	0.079	1.3E-02	**6.7E-16**	HDL
10	114,754,784	*TCF7L2*	rs35198068	T	C	0.70	**−0.066**	**2.5E-11**	−0.001	9.8E-01	−0.034	2.7E-01	**5.0E-19**	T2D
11	61,593,816	*FADS2*	rs174568	C	T	0.66	0.030	1.6E-03	**−0.244**	**2.8E-08**	−0.019	5.3E-01	**5.0E-39**	TG
11	116,648,917	*ZNF259*	rs964184	G	C	0.15	0.012	3.5E-01	**0.622**	**2.2E-34**	0.199	1.1E-07	**3.5E-155**	TG, HDL
15	58,679,807	*LIPC*	15:58679807	CAGA	C	0.65	**−0.093**	**3.1E-22**	−0.122	6.0E-03	−0.084	4.2E-03	NA	HDL
15	91,420,973	*FURIN*	rs1573643	T	C	0.67	**−0.061**	**2.5E-10**	−0.027	5.6E-01	−0.005	8.6E-01	1.5E-05	Blood pressure
16	57,006,590	*CETP*	rs7499892	C	T	0.80	**0.160**	**2.8E-44**	0.078	1.5E-01	0.060	9.1E-02	**1.8E-09**	HDL
16	75,456,304	*CFDP1*	rs12934767	T	C	0.42	**−0.052**	**1.7E-08**	−0.055	2.2E-01	0.013	6.5E-01	NA	CAD
19	45,413,233	*APOE*	rs1065853	G	T	0.94	0.030	1.1E-01	**−0.583**	**4.5E-16**	0.260	3.0E-05	**0**	LDL, HDL, TG

The variant with smallest P in each locus is shown for each drug. Genome-wide significant association (P <5e-8) in bold.

* Statin medication use GWAS in FinnGen.

** Looked up in https://hugeamp.org/ and https://atlas.ctglab.nl/PheWAS

**Table 4.** Significant loci for GWAS of hypertension drug choice

							Diuretic		β-blocker		Calcium channel blocker		RAS inhibitor		FinnGen*	
Chr	Position_hg19	Nearby gene	SNP ID	Effect allele	Other allele	Effect allele frequency	log_OR	P	log_OR	P	log_OR	P	log_OR	P	P	Other associated traits**
						
1	10,796,866	*CASZ1*	rs880315	T	C	0.65	−0.047	3.0E-06	−0.033	2.9E-03	**−0.083**	**8.1E-16**	−0.043	4.6E-07	**1.9E-34**	Blood pressure
1	113,052,923	*WNT2B*	rs11102485	C	G	0.92	−0.066	1.3E-04	−0.046	1.7E-02	**−0.128**	**3.8E-13**	−0.044	3.1E-03	**6.6E-45**	Blood pressure
1	113,265,881	*WNT2B*	rs77724279	C	T	0.95	**−0.123**	**2.8E-08**	0.013	6.1E-01	−0.082	3.2E-04	−0.058	2.2E-03	**2.1E-22**	Blood pressure
2	26,922,062	*KCNK3*	rs1314982	G	A	0.27	**0.071**	**4.6E-11**	0.040	8.6E-04	**0.066**	**2.6E-09**	0.038	3.6E-05	NA	Blood pressure
2	26,932,887	*KCNK3*	rs1275922	A	G	0.26	**0.070**	**9.5E-11**	0.043	3.8E-04	**0.068**	**1.1E-09**	0.037	5.0E-05	**1.2E-39**	Blood pressure
4	81,202,048	*FGF5*	rs36034102	G	T	0.72	−0.007	5.4E-01	−0.039	1.1E-03	−0.026	1.9E-02	**−0.064**	**2.1E-12**	**2.4E-40**	Blood pressure
6	127,187,921	*RSPO3*	rs55994889	C	CA	0.56	−0.050	2.0E-07	−0.035	1.3E-03	−0.047	2.5E-06	**−0.045**	**4.9E-08**	NA	Bone mineral density
6	160,816,409	*LPA*	rs1510226	T	C	0.98	0.015	6.6E-01	**−0.205**	**2.6E-08**	−0.035	3.2E-01	−0.050	8.3E-02	1.0E-04	Lipoprotein(a), LDL
7	27,236,559	*EVX1*	rs115525024	T	G	0.07	**−0.131**	**9.1E-12**	−0.044	3.3E-02	−0.092	3.1E-06	−0.066	3.9E-05	**1.3E-33**	Blood pressure
7	27,319,039	*EVX1*	rs28451436	G	A	0.90	−0.076	1.0E-06	−0.029	9.2E-02	**−0.099**	**6.3E-10**	−0.056	2.4E-05	**1.3E-16**	Blood pressure
7	150,690,176	*NOS3*	rs3918226	C	T	0.91	−0.089	2.0E-07	−0.086	4.6E-06	−0.077	1.1E-05	**−0.091**	**3.3E-10**	**1.9E-49**	Blood pressure
9	22,103,183	*CDKN2B*	rs1537372	G	T	0.57	−0.007	4.8E-01	**−0.073**	**1.3E-11**	−0.012	2.4E-01	−0.028	5.3E-04	NA	CAD
11	1,885,025	*LSP1*	rs673791	C	G	0.37	**−0.067**	**1.2E-11**	−0.011	3.1E-01	−0.054	1.4E-07	−0.045	7.0E-08	**1.3E-12**	Blood pressure
11	1,887,068	*LSP1*	rs569550	T	G	0.60	**−0.060**	**7.9E-10**	−0.028	1.0E-02	**−0.063**	**4.2E-10**	**−0.048**	**7.8E-09**	**1.1E-23**	Blood pressure
11	1,890,990	*LSP1*	rs592373	G	A	0.36	**−0.060**	**1.6E-09**	−0.019	8.8E-02	−0.053	2.0E-07	**−0.049**	**5.7E-09**	**2.1E-14**	Blood pressure
11	100,610,546	*ARHGAP42*	rs604723	T	C	0.27	−0.019	8.3E-02	−0.024	4.4E-02	−0.038	8.0E-04	**−0.059**	**1.1E-10**	**2.6E-37**	Blood pressure
12	111,884,608	*SH2B3*	rs3184504	T	C	0.49	0.031	1.1E-03	**0.077**	**5.0E-13**	0.012	2.2E-01	**0.050**	**6.8E-10**	**7.7E-43**	Blood pressure
12	115,553,115	*RP11–116D17.1*	rs35441	C	T	0.63	**0.055**	**3.1E-08**	0.030	6.0E-03	0.047	4.1E-06	0.044	1.5E-07	**4.0E-11**	Blood pressure
12	117,703,762	*NOS1*	12:117703762 AT A	AT	A	0.34	−0.005	6.3E-01	0.007	5.2E-01	**0.059**	**2.5E-08**	−0.012	1.6E-01	NA	
13	22,295,868	*FGF9*	rs620124	G	C	0.33	**−0.061**	**3.6E-09**	−0.028	1.3E-02	**−0.063**	**2.3E-09**	−0.019	2.7E-02	**9.5E-10**	Blood pressure
13	22,297,802	*FGF9*	rs9552473	T	G	0.34	**−0.063**	**7.7E-10**	−0.033	3.2E-03	**−0.062**	**3.7E-09**	−0.021	1.4E-02	**5.0E-10**	Blood pressure
13	32,187,858	*RXFP2*	rs12876275	G	T	0.49	−0.042	1.0E-05	−0.030	4.1E-03	**−0.071**	**5.9E-13**	−0.020	1.1E-02	3.1E-01	Blood pressure

The variant with smallest P in each locus is shown for each drug. Genome-wide significant association (P <5e-8) in bold.

* Antihypertensive medication use GWAS in FinnGen.

** Looked up in https://hugeamp.org/ and https://atlas.ctglab.nl/PheWAS

**Table 5A.** Prediction of dyslipidemia drug choice

	AUC-ROC in UK Biobank, EUR				AUC-ROC in All of Us, EUR		AUC-ROC in UK Biobank, SAS	
Drug	PGS	Basic	Combined	P, AUC_basic_ < AUC_combined_ *	PGS	P, AUC_PGS_ >0.5 *	PGS	P, AUC_PGS_ >0.5 *
			
Statin	0.555	0.694	0.702	1.5E-17	0.516	6.6E-16	0.572	1.9E-13
Fibrate	0.621	0.619	0.675	4.3E-08	0.614	1.3E-121	0.620	4.1E-03
Ezetimibe	0.550	0.622	0.633	4.0E-03	0.549	7.3E-27	0.554	5.5E-02

**Table 5B.** Prediction of hypertension drug choice

	AUC-ROC in UK Biobank, EUR				AUC-ROC in All of Us, EUR		AUC-ROC in UK Biobank, SAS	
Drug	PGS	Basic	Combined	P, AUC_basic_ < AUC_combined_ *	PGS	P, AUC_PGS_ >0.5 *	PGS	P, AUC_PGS_ >0.5 *
			
Diuretic	0.524	0.649	0.651	1.2E-04	0.503	0.07	0.517	0.11
β-blocker	0.533	0.614	0.620	1.1E-06	0.507	6.0E-04	0.532	1.1E-02
Calcium channel blocker	0.534	0.624	0.630	4.8E-08	0.516	4.3E-14	0.508	0.25
RAS-acting agents	0.534	0.617	0.624	5.5E-11	0.521	4.6E-24	0.549	2.7E-06

EUR, European descent; SAS, South Asian descent;

* alternative hypothesis

## Data Availability

Code files for analyses in this manuscript are available from https://github.com/fumi-github/drug_selection

## References

[1] Nelson MR, Johnson T, Warren L The genetics of drug efficacy: opportunities and challenges. Nat. Rev. Genet. 17(4), 197–206 (2016). 26972588 10.1038/nrg.2016.12

[2] Postmus I, Trompet S, Deshmukh HA Pharmacogenetic meta-analysis of genome-wide association studies of LDL cholesterol response to statins. Nat. Commun. 5(1), 5068 (2014). 25350695 10.1038/ncomms6068 PMC4220464

[3] Kanoni S, Graham SE, Wang Y Implicating genes, pleiotropy, and sexual dimorphism at blood lipid loci through multi-ancestry meta-analysis. Genome Biol 23(1), 268 (2022). 36575460 10.1186/s13059-022-02837-1 PMC9793579

[4] Gong Y, McDonough CW, Wang Z Hypertension Susceptibility Loci and Blood Pressure Response to Antihypertensives. Circ.: Cardiovasc. Genet. 5(6), 686–691 (2012). 23087401 10.1161/CIRCGENETICS.112.964080 PMC3529147

[5] Salvi E, Wang Z, Rizzi F Genome-Wide and Gene-Based Meta-Analyses Identify Novel Loci Influencing Blood Pressure Response to Hydrochlorothiazide. Hypertension 69(1), 51–59 (2017). 27802415 10.1161/HYPERTENSIONAHA.116.08267 PMC5145728

[6] Wu Y, Byrne EM, Zheng Z Genome-wide association study of medication-use and associated disease in the UK Biobank. Nat. Commun. 10(1), 1891 (2019). 31015401 10.1038/s41467-019-09572-5 PMC6478889

[7] Sudlow C, Gallacher J, Allen N UK biobank: an open access resource for identifying the causes of a wide range of complex diseases of middle and old age. PLoS medicine 12(3), e1001779 (2015). 25826379 10.1371/journal.pmed.1001779 PMC4380465

[8] Tsiara S, Elisaf M, Mikhailidis DP. Early vascular benefits of statin therapy. Curr. Méd. Res. Opin. 19(6), 540–556 (2003). 14594527 10.1185/030079903125002225

[9] Lasserson DS, Buclin T, Glasziou P. How quickly should we titrate antihypertensive medication? Systematic review modelling blood pressure response from trial data. Heart 97(21), 1771 (2011). 21586424 10.1136/hrt.2010.221473

[10] Detection E and Treatment of High Blood Cholesterol in Adults Expert Panel on. Executive Summary of the Third Report of the National Cholesterol Education Program (NCEP) Expert Panel on Detection, Evaluation, and Treatment of High Blood Cholesterol in Adults (Adult Treatment Panel III). JAMA 285(19), 2486–2497 (2001). 11368702 10.1001/jama.285.19.2486

[11] Unger T, Borghi C, Charchar F 2020 International Society of Hypertension Global Hypertension Practice Guidelines. Hypertension 75(6), 1334–1357 (2020). 32370572 10.1161/HYPERTENSIONAHA.120.15026

[12] Bycroft C, Freeman C, Petkova D The UK Biobank resource with deep phenotyping and genomic data. Nature 562(7726), 203–209 (2018). 30305743 10.1038/s41586-018-0579-z PMC6786975

[13] Investigators A of URP, Denny JC, Rutter JL The “All of Us” Research Program. N. Engl. J. Med. 381(7), 668–676 (2019). 31412182 10.1056/NEJMsr1809937 PMC8291101

[14] Investigators TA of URPG, Group MW, Bick AG Genomic data in the All of Us Research Program. Nature 627(8003), 340–346 (2024). 38374255 10.1038/s41586-023-06957-x PMC10937371

[15] Mbatchou J, Barnard L, Backman J Computationally efficient whole-genome regression for quantitative and binary traits. Nature Genetics 1–20 (2021). 34017140 10.1038/s41588-021-00870-7

[16] Willer CJ, Li Y, Abecasis GR. METAL: fast and efficient meta-analysis of genomewide association scans. Bioinformatics 26(17), 2190–2191 (2010). 20616382 10.1093/bioinformatics/btq340 PMC2922887

[17] Turner SD. qqman: an R package for visualizing GWAS results using Q-Q and manhattan plots. J. Open Source Softw. 3(25), 731 (2018).

[18] Kurki MI, Karjalainen J, Palta P FinnGen provides genetic insights from a well-phenotyped isolated population. Nature 613(7944), 508–518 (2023). 36653562 10.1038/s41586-022-05473-8 PMC9849126

[19] Bulik-Sullivan B, Finucane HK, Anttila V An atlas of genetic correlations across human diseases and traits. Nature Genetics 47(11), 1236–1241 (2015). 26414676 10.1038/ng.3406 PMC4797329

[20] Martin J, Khramtsova EA, Goleva SB Examining Sex-Differentiated Genetic Effects Across Neuropsychiatric and Behavioral Traits. Biol. Psychiatry 89(12), 1127–1137 (2021). 33648717 10.1016/j.biopsych.2020.12.024 PMC8163257

[21] Consortium GLG, Willer CJ, Schmidt EM Discovery and refinement of loci associated with lipid levels. Nature Genetics 45(11), 1274–1283 (2013). 24097068 10.1038/ng.2797 PMC3838666

[22] Surendran P, Drenos F, Young R Trans-ancestry meta-analyses identify rare and common variants associated with blood pressure and hypertension. Nature Genetics 48(10), 1151–1161 (2016). 27618447 10.1038/ng.3654 PMC5056636

[23] Liu C, Kraja AT, Smith JA Meta-analysis identifies common and rare variants influencing blood pressure and overlapping with metabolic trait loci. Nature Genetics 48(10), 1162–1170 (2016). 27618448 10.1038/ng.3660 PMC5320952

[24] Hoffmann TJ, Ehret GB, Nandakumar P Genome-wide association analyses using electronic health records identify new loci influencing blood pressure variation. Nature Genetics (2016). 10.1038/ng.3715 PMC5370207 27841878

[25] Scott RA, Scott LJ, Mägi R An Expanded Genome-Wide Association Study of Type 2 Diabetes in Europeans. Diabetes 66(11), 2888–2902 (2017). 28566273 10.2337/db16-1253 PMC5652602

[26] Locke AE, Kahali B, Berndt SI Genetic studies of body mass index yield new insights for obesity biology. Nature 518(7538), 197–206 (2015). 25673413 10.1038/nature14177 PMC4382211

[27] Cardiogram Consortium. A comprehensive 1000 Genomes-based genome-wide association meta-analysis of coronary artery disease. Nature Genetics 47(10), 1121–1130 (2015). 26343387 10.1038/ng.3396 PMC4589895

[28] Sheng X, Xia L, Cahoon JL Inverted genomic regions between reference genome builds in humans impact imputation accuracy and decrease the power of association testing. Hum. Genet. Genom. Adv. 4(1), 100159 (2023). 10.1016/j.xhgg.2022.100159 PMC9709082 36465187

[29] Sadler MC, Apostolov A, Cevallos C Leveraging large-scale biobank EHRs to enhance pharmacogenetics of cardiometabolic disease medications. Nat. Commun. 16(1), 2913 (2025). 40133288 10.1038/s41467-025-58152-3 PMC11937416

[30] Chasman DI, Giulianini F, MacFadyen J, Barratt BJ, Nyberg F, Ridker PM. Genetic Determinants of Statin-Induced Low-Density Lipoprotein Cholesterol Reduction. Circ.: Cardiovasc. Genet. 5(2), 257–264 (2012). 22331829 10.1161/CIRCGENETICS.111.961144

[31] Hopewell JC, Parish S, Offer A Impact of common genetic variation on response to simvastatin therapy among 18 705 participants in the Heart Protection Study. Eur. Hear. J. 34(13), 982–992 (2013). 10.1093/eurheartj/ehs344 PMC3612775 23100282

[32] Oni-Orisan A, Haldar T, Ranatunga DK The impact of adjusting for baseline in pharmacogenomic genome-wide association studies of quantitative change. npj Genom. Med. 5(1), 1 (2020). 31969989 10.1038/s41525-019-0109-4 PMC6965183

[33] Deshmukh HA, Colhoun HM, Johnson T Genome-wide association study of genetic determinants of LDL-c response to atorvastatin therapy: importance of Lp(a). J. Lipid Res. 53(5), 1000–1011 (2012). 22368281 10.1194/jlr.P021113 PMC3329377

[34] Yanez ND, Kronmal RA, Shemanski LR. The effects of measurement error in response variables and tests of association of explanatory variables in change models. Stat. Med. 17(22), 2597–2606 (1998). 9839350 10.1002/(sici)1097-0258(19981130)17:22<2597::aid-sim940>3.0.co;2-g

[35] McArdle PF, Whitcomb BW. Improper Adjustment for Baseline in Genetic Association Studies of Change in Phenotype. Hum. Hered. 67(3), 176–182 (2009). 19077436 10.1159/000181156 PMC2868920

[36] Kloet AD de Krause EG, Woods SC. The renin angiotensin system and the metabolic syndrome. Physiol. Behav. 100(5), 525–534 (2010). 20381510 10.1016/j.physbeh.2010.03.018 PMC2886177

[37] Udler MS, McCarthy MI, Florez JC, Mahajan A. Genetic Risk Scores for Diabetes Diagnosis and Precision Medicine. Endocr. Rev. 40(6), 1500–1520 (2019). 31322649 10.1210/er.2019-00088 PMC6760294

[38] Suzuki K, Hatzikotoulas K, Southam L Genetic drivers of heterogeneity in type 2 diabetes pathophysiology. Nature 627(8003), 347–357 (2024). 38374256 10.1038/s41586-024-07019-6 PMC10937372

[39] Siemens A, Anderson SJ, Rassekh SR, Ross CJD, Carleton BC. A Systematic Review of Polygenic Models for Predicting Drug Outcomes. J. Pers. Med. 12(9), 1394 (2022). 36143179 10.3390/jpm12091394 PMC9505711

[40] Johnson D, Wilke MAP, Lyle SM A Systematic Review and Analysis of the Use of Polygenic Scores in Pharmacogenomics. Clin. Pharmacol. Ther. 111(4), 919–930 (2022). 34953075 10.1002/cpt.2520

