# Supplementary material for: Pharmacogenomic predictors of drug response and choice in dyslipidemia and hypertension
Source: medRxiv. 2026 Jan 30:2026.01.28.26345024. Preprint. [Version 1] doi: 10.64898/2026.01.28.26345024 (PMC12870588; doi:10.64898/2026.01.28.26345024)
Supplement: Supplement 1 [file NIHPP2026.01.28.26345024v1-supplement-1.pdf]

medRxiv preprint doi: https://doi.org/10.64898/2026.01.28.26345024; this version posted January 30, 2026. The copyright holder for this preprint (which was not certified by peer review) is the author/funder, who has granted medRxiv a license to display the preprint in perpetuity.
It is made available under a CC-BY 4.0 International license .

Supplementary Figure 1

Manhattan plots for GWAS of drug-induced biomarker response after variance-stabilizing Box-Cox transformation. Variants are plotted as points, with chromosomal position along the horizontal axis, and $-\log_{10}$(P-value) along the vertical axis.

Supplementary Figure 2

Performance of drug-specific PGS for dyslipidemia (**A**) and hypertension (**B**). Left panels illustrate cross-comparisons of drug choice frequencies across PGS deciles trained for different medications in the UK Biobank EUR subjects. Rows represent the tested drugs, and columns represent the drugs used for PGS training. Bar heights indicate the proportion of subjects prescribed each drug across PGS deciles, with error bars indicating the standard deviation from cross-validation trials. The tables on the right summarize the corresponding Area Under the Receiver Operating Characteristic Curve (AUC-ROC) for each combination. For each tested drug (row), the model with the highest AUC-ROC is highlighted in bold font. This analysis demonstrates the specificity of each drug's PGS for predicting its own intended prescription pattern.

medRxiv preprint doi: https://doi.org/10.64898/2026.01.28.26345024; this version posted January 30, 2026. The copyright holder for this preprint (which was not certified by peer review) is the author/funder, who has granted medRxiv a license to display the preprint in perpetuity.
It is made available under a CC-BY 4.0 International license .

Supplementary Figure 1

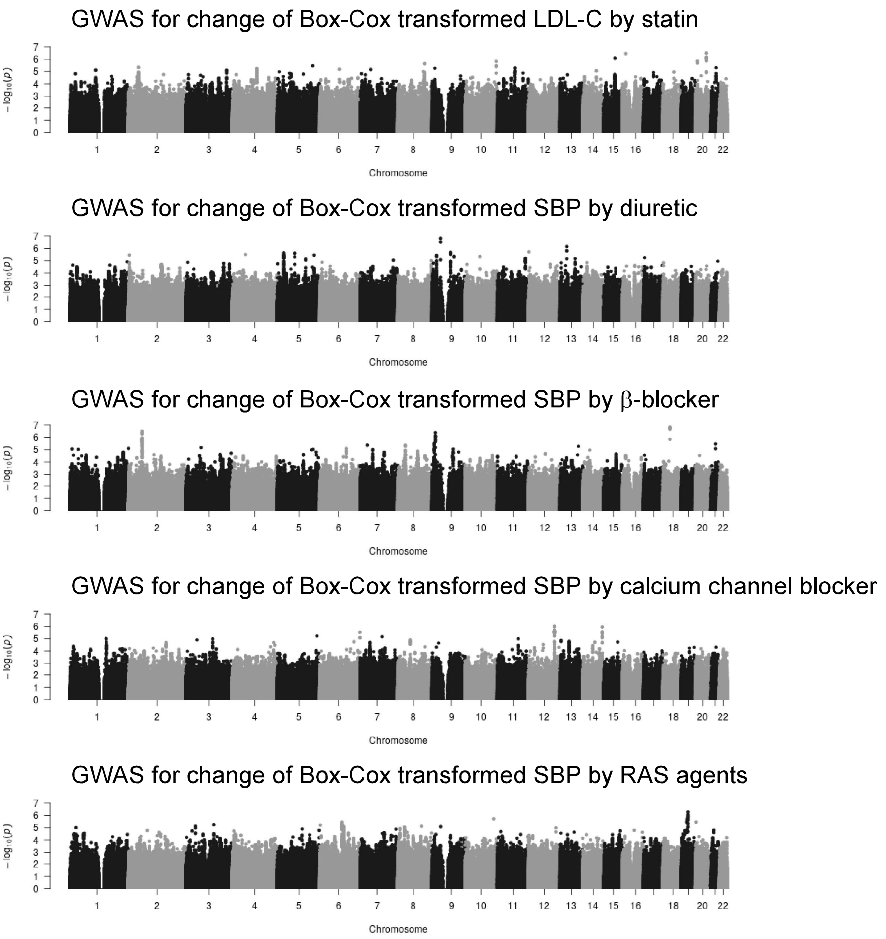

Manhattan plots for GWAS of drug-induced biomarker response after variance-stabilizing Box-Cox transformation. Variants are plotted as points, with chromosomal position along the horizontal axis, and -$\log_{10}$(P-value) along the vertical axis.

medRxiv preprint doi: https://doi.org/10.64898/2026.01.28.26345024; this version posted January 30, 2026. The copyright holder for this preprint (which was not certified by peer review) is the author/funder, who has granted medRxiv a license to display the preprint in perpetuity.
It is made available under a CC-BY 4.0 International license .

Supplementary Figure 2

A. Dyslipidemia drugs

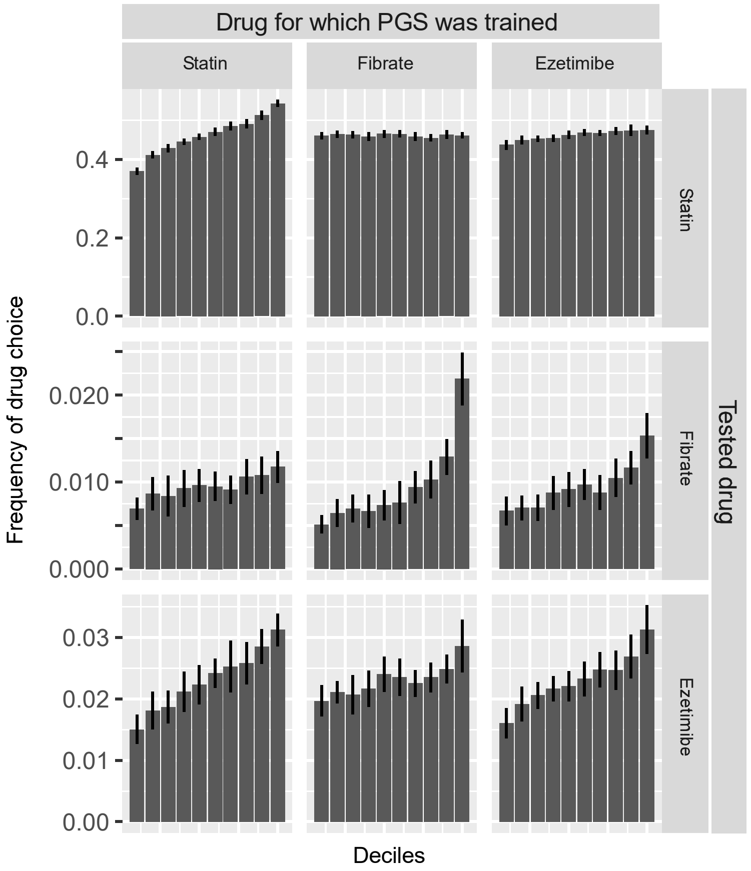

AUC-ROC

**0.555**	0.499	0.513
0.537	**0.621**	0.570
**0.560**	0.528	0.550

B. Hypertension drugs

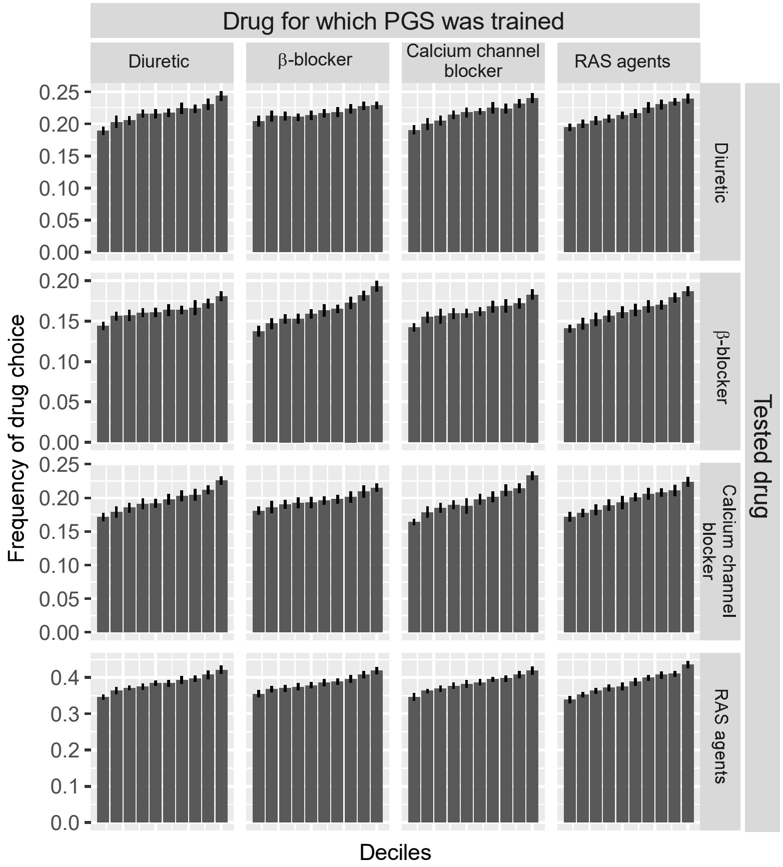

AUC-ROC

0.524	0.512	0.524	**0.524**
0.519	**0.533**	0.521	0.524
0.528	0.518	**0.534**	0.529
0.525	0.522	0.525	**0.534**

Performance of drug-specific PGS for dyslipidemia (**A**) and hypertension (**B**).
Left panels illustrate cross-comparisons of drug choice frequencies across PGS deciles trained for different medications in the UK Biobank EUR subjects. Rows represent the tested drugs, and columns represent the drugs used for PGS training. Bar heights indicate the proportion of subjects prescribed each drug across PGS deciles, with error bars indicating the standard deviation from cross-validation trials. The tables on the right summarize the corresponding Area Under the Receiver Operating Characteristic Curve (AUC-ROC) for each combination. For each tested drug (row), the model with the highest AUC-ROC is highlighted in bold font. This analysis demonstrates the specificity of each drug's PGS for predicting its own intended prescription pattern.
